# Cross-Cultural Care: The Experiences of Pediatric Nurses in Providing Culturally Sensitive Care to Children and Their Families

**DOI:** 10.3390/healthcare14172784

**Published:** 2026-09-01

**Authors:** Abdulaziz M. Alodhailah, Majed M. Aljabri, Abdullah Alharbi, Bader M. Almutairy, Faihan F. Alshaibany, Mohammed Almutairi

**Affiliations:** 1 Department of Medical-Surgical Nursing, College of Nursing, King Saud University, Riyadh 11451, Saudi Arabia; 2 Department of Community and Mental Health Nursing, College of Nursing, King Saud University, Riyadh 11451, Saudi Arabia; 3 Department of Nursing Administration and Education, College of Nursing, King Saud University, Riyadh 11451, Saudi Arabia

**Keywords:** cultural competence, pediatric nursing, culturally sensitive care, qualitative research, Saudi Arabia, cross-cultural care

## Abstract

Background: Saudi Arabia’s healthcare system serves an increasingly diverse patient population, yet limited qualitative evidence exists regarding how pediatric nurses navigate cultural complexity in their daily practice. Understanding nurses’ day-to-day experiences of cross-cultural care is essential for strengthening culturally sensitive pediatric services. Purpose: This study explored the experiences of pediatric nurses in providing culturally sensitive care to children and their families in Riyadh, Saudi Arabia. Methods: A qualitative descriptive study was conducted in pediatric units of tertiary hospitals in Riyadh City, Saudi Arabia. Eighteen pediatric nurses participated in semi-structured interviews. Data were analyzed using Braun and Clarke’s reflexive thematic analysis, and reporting followed the Consolidated Criteria for Reporting Qualitative Research (COREQ) guidelines. Results: Four themes emerged: navigating cultural landscapes, describing nurses’ encounters with diverse cultural beliefs and health practices; communication as a bridge and a barrier, reflecting language challenges and strategies for meaningful engagement; balancing professional standards with cultural respect, highlighting the tension between evidence-based care and cultural accommodation; and the emotional labor of cross-cultural caregiving, capturing the personal toll and resilience involved in culturally diverse practice. Conclusions: Pediatric nurses engage in complex interpretive, relational, and emotional work when providing culturally sensitive care. Institutional support through cultural competence training, multilingual resources, and organizational policies is essential for enhancing culturally responsive pediatric nursing practice in diverse healthcare settings.

## 1. Introduction

Globalization and international labor migration have transformed the demographic composition of patient populations in healthcare systems worldwide [1]. In the Arabian Gulf region, and particularly in Saudi Arabia, rapid economic development under Vision 2030 has attracted a multinational workforce, resulting in an exceptionally diverse resident population that includes families from South Asia, Southeast Asia, Africa, the Middle East, and Western countries [2]. This cultural heterogeneity presents both opportunities and challenges for healthcare delivery, especially in pediatric settings where care extends beyond the individual child to encompass the family unit as a whole [3].

Culturally sensitive care, defined as the recognition, respect, and integration of patients’ cultural values, beliefs, and practices into clinical decision-making, has been widely endorsed as a cornerstone of patient-centered healthcare [4,5]. In pediatric nursing, cultural sensitivity assumes particular importance because children’s health-related behaviours, illness perceptions, and care preferences are profoundly shaped by familial and cultural contexts [6]. Parents and caregivers serve as primary decision-makers and mediators between the healthcare system and the child, making culturally attuned communication and family engagement essential to effective care [7].

Despite growing recognition of cultural competence as a professional imperative, research suggests that nurses frequently encounter barriers to providing culturally sensitive care. These barriers include language differences, unfamiliarity with cultural health practices, conflicting beliefs about illness causation and treatment, and institutional constraints that limit the time and resources available for culturally responsive engagement [8,9]. In pediatric settings, additional complexities arise from the developmental needs of children, the emotional intensity of family-centered care, and the varied expectations of families regarding parental involvement in clinical decision-making [10].

In Saudi Arabia, the healthcare workforce is predominantly composed of expatriate nurses from diverse national backgrounds, creating a unique cross-cultural dynamic in which both patients and nurses bring distinct cultural perspectives to the care encounter [11]. This bidirectional cultural diversity introduces interpretive challenges that may affect therapeutic relationships, treatment adherence, and family satisfaction with care [12]. While quantitative studies have examined cultural competence levels and associated factors among nurses in Saudi Arabia [13,14], limited qualitative research has explored how pediatric nurses experience and navigate the complexities of providing culturally sensitive care in practice. Recent qualitative work has examined nurse-parent communication in culturally sensitive pediatric care [15], pediatric nurses’ cultural competence in health visits with children of foreign background [16] and the integration of culturally and linguistically diverse nurses into multinational healthcare workforces [17]. The cross-cultural leadership capacity of nurse managers is a related but distinct factor: nurse leaders shape unit-level norms, staffing, and orientation practices, and prior work on integrating culturally and linguistically diverse nurses suggests that leadership behavior can either support or constrain frontline nurses’ ability to deliver culturally sensitive care [17], but these two bodies of literature have largely been considered separately. The present study addresses this gap by examining the bidirectional cultural dynamic of the Saudi context, in which an internationally diverse nursing workforce provides care to an equally diverse pediatric population, within a single interpretive framework.

Understanding nurses’ experiences is essential for identifying practical strategies to enhance culturally responsive care delivery. Qualitative inquiry enables exploration of the nuances of cross-cultural encounters, including the tacit knowledge, emotional dimensions, and relational dynamics that quantitative measures may not capture [18]. By examining how pediatric nurses perceive, interpret, and respond to cultural diversity in their daily practice, this study seeks to generate contextualized insights that can inform educational interventions, organizational policies, and clinical practice guidelines.

The aim of this study was therefore to explore the experiences of pediatric nurses in providing culturally sensitive care to children and their families in Riyadh, Saudi Arabia. Specifically, the study addressed the following research questions:

1. How do pediatric nurses describe their experiences of providing care to culturally diverse children and families?
2. What challenges and facilitators do nurses encounter in delivering culturally sensitive pediatric care?
3. How do nurses negotiate cultural differences while maintaining professional standards of care?

## 2. Materials and Methods

### 2.1. Design

This study employed a qualitative descriptive design to explore how pediatric nurses experience providing culturally sensitive care to children and their families. Qualitative description, as articulated by Sandelowski [19], seeks to produce a comprehensive account of events in everyday language, staying close to participants’ accounts while allowing for interpretive engagement with the data. This approach was considered appropriate because the study aimed to capture the practical, relational, and emotional dimensions of cross-cultural care as experienced by nurses in clinical practice. The specific analytic procedures used to move from the interviews to the themes reported below are described in full in Section 2.7, to avoid duplicating that account here. Data were analyzed using Braun and Clarke’s reflexive thematic analysis [20], and reporting followed the Consolidated Criteria for Reporting Qualitative Research (COREQ) guidelines [21].

### 2.2. Setting

The study was conducted in pediatric inpatient units within tertiary healthcare hospitals in Riyadh City, Saudi Arabia. These hospitals serve as major referral centers and provide comprehensive pediatric services, including general pediatrics, pediatric surgery, pediatric intensive care, neonatal care, and subspecialty clinics. The patient population represents considerable cultural diversity, including Saudi nationals and expatriate families from South Asia, Southeast Asia, Africa, and the Middle East. The nursing workforce is similarly diverse, comprising nurses from the Philippines, India, Saudi Arabia, Egypt, Jordan, and other countries. This dual cultural diversity, among both patients and providers, provided an appropriate context for exploring cross-cultural care experiences.

### 2.3. Participants and Sampling

Participants were registered nurses currently working in pediatric inpatient units with direct clinical responsibility for the care of children and their families. Inclusion criteria were: (1) a minimum of one year of experience in pediatric nursing; (2) current employment in a pediatric ward, pediatric intensive care unit, or neonatal unit; and (3) experience caring for culturally diverse patient populations. Nurses in exclusively administrative or supervisory roles without direct patient contact were excluded.

Purposive sampling was used to recruit participants with diverse demographic and professional characteristics, including variation in nationality, years of experience, educational background, and unit type. This strategy aimed to capture a breadth of cross-cultural care experiences. Recruitment continued until data richness and thematic sufficiency were achieved [22], with an anticipated sample of 15–20 participants. Sufficiency was judged through iterative comparison of newly generated codes against the developing coding framework, and recruitment concluded when subsequent interviews reinforced rather than expanded this framework, consistent with an information-power rather than a purely numerical approach to sample adequacy [23]. The anticipated range of 15–20 was set with reference to the specificity of the study aim, the relative homogeneity of the participant group (registered pediatric nurses within a defined set of tertiary hospitals), and comparable prior qualitative nursing studies of similar scope, rather than derived from a fixed a priori formula [22,23]. Of the nurses approached, three declined to participate, citing scheduling conflicts.

### 2.4. Recruitment

Following institutional approvals, nursing managers in participating pediatric units were informed about the study and assisted in distributing invitation emails to eligible nurses. Interested participants contacted the research team directly to minimize potential perceived managerial pressure. Written information sheets describing the study purpose, procedures, confidentiality, voluntary nature of participation, and the right to withdraw were provided before obtaining informed consent.

### 2.5. Data Collection

Data were collected through individual, semi-structured, in-depth interviews conducted between April and May 2026. Interviews were held in a private room within the hospital premises or via secure video conferencing when face-to-face meetings were not feasible. Each interview began with a restatement of the study purpose and confidentiality procedures, proceeded through the semi-structured open-ended questions and probes described below, and closed with an opportunity for participants to add information they felt had not been covered. Each interview lasted between 40 and 68 min (mean 49.7 ± 7.2 min). No repeat interviews were conducted; each participant was interviewed once.

An interview guide was developed specifically for this study based on existing literature on cultural competence, transcultural nursing theory, and pediatric family-centered care. The English version of the semi-structured interview guide is provided as Supplementary File S1. Questions were open-ended and exploratory, designed to elicit detailed descriptions of cross-cultural encounters. Example prompts included the following:

- “Can you describe a situation where cultural differences influenced how you provided care to a child and their family?”
- “What challenges have you faced when caring for families from different cultural backgrounds?”
- “How do you manage situations where cultural practices conflict with medical recommendations?”
- “What has helped you provide better care to families from different cultures?”

Probing questions were used to elicit clarification and encourage deeper reflection. The interview guide was refined iteratively as data collection progressed. The guide was pilot-tested with four participants who were not included in the final analytic sample, and refined accordingly prior to data collection with the 18 participants reported here. All interviews were audio-recorded with participant consent and transcribed verbatim. Interviews conducted in Arabic were transcribed and translated into English by a bilingual member of the research team; to verify translation accuracy, 10% of the translated transcripts, randomly selected, underwent back-translation into Arabic, which was compared against the original recordings and found to be consistent. Field notes documenting contextual observations and initial analytic reflections were maintained throughout data collection.

### 2.6. Researcher Characteristics and Reflexivity

The interviews were conducted by a member of the research team who is a healthcare professional with doctoral-level qualifications and substantial clinical and qualitative research experience. This professional background provided familiarity with pediatric healthcare settings and facilitated meaningful engagement with participants while maintaining a respectful and professional interview environment. The interviewer had no direct supervisory or managerial relationship with any of the participants, thereby reducing the likelihood of coercion or role-related influence.

The research team comprised both male and female healthcare professionals with doctoral-level qualifications and complementary expertise in clinical practice, qualitative research, and nursing scholarship. The diversity of the research team contributed multiple professional and cultural perspectives during data interpretation, helping to minimize the influence of any single researcher’s assumptions or preconceptions.

Throughout the study, the researchers adopted a reflexive approach by continually reflecting on how their professional backgrounds, prior clinical experiences, and perspectives could shape data collection and interpretation. Reflexive discussions were conducted regularly during data analysis to critically examine emerging codes, themes, and interpretations. Coding decisions and theme development were reviewed collaboratively by the research team until consensus was reached, ensuring that the findings remained grounded in participants’ accounts rather than researchers’ expectations. These strategies enhanced the credibility, confirmability, and trustworthiness of the findings and are consistent with established qualitative research reporting standards, including COREQ [21] and SRQR [24].

### 2.7. Data Analysis

Data analysis followed Braun and Clarke’s six-phase reflexive thematic analysis framework [20]: (1) familiarization with the data through repeated reading of transcripts; (2) generation of initial codes across the entire dataset; (3) searching for themes by collating codes into potential thematic groupings; (4) reviewing themes against coded extracts and the full dataset; (5) defining and naming themes to capture their essential meaning; and (6) producing the analytic report with selected illustrative quotations. Reflexive thematic analysis was selected, rather than a more structured content-analytic or purely descriptive coding approach, because it is compatible with the interpretive orientation of qualitative description while still providing a systematic, auditable procedure for moving from coded data to conceptually coherent themes [20].

Analysis was conducted inductively, allowing themes to emerge from the data rather than being imposed through predetermined frameworks. Consistent with Braun and Clarke’s conceptualization of reflexive thematic analysis, themes were developed as patterns of shared meaning organized around a central interpretive concept, rather than as topic summaries or descriptive categories of the data; codes were therefore retained as themes only where they captured a shared underlying meaning across multiple participants’ accounts, not merely a common topic of discussion. Coding was performed manually by two authors of the research team, without the use of qualitative data analysis software, to organize transcripts and facilitate systematic comparison. For example, initial descriptive codes such as “family attributes illness to non-biomedical causes” and “declines treatment based on traditional belief” were collated into a broader code labeled “coexistence of biomedical and cultural explanatory models”; this code was subsequently grouped with related codes concerning family hierarchy and decision-making authority under the overarching theme Navigating Cultural Landscapes, reflecting the shared underlying pattern of nurses interpreting and working within families’ own explanatory frameworks rather than displacing them. Analytic decisions were documented in an audit trail comprising coding notes, theme development memos, and team discussion records. During the final phase of analysis, the research team additionally examined the relationships among the four generated themes and synthesized these relationships into an integrative conceptual model, presented in Section 3.3; this model therefore represents a further inductive step built directly on the coded data and themes described here, rather than a framework introduced independently of the analysis. Discrepancies in code and theme labeling between the two coding authors, identified through systematic comparison of their independently produced coding outputs, were resolved through discussion and, where necessary, joint re-examination of the original transcripts and field notes until consensus was reached.

### 2.8. Rigor and Trustworthiness

Rigor was established through adherence to Lincoln and Guba’s criteria of credibility, dependability, confirmability, and transferability [25]. Credibility was enhanced through prolonged engagement with the data, iterative questioning during interviews, and peer debriefing within the research team. Selected participants were invited to review a summary of emerging themes to verify resonance with their experiences. Dependability was supported by maintaining a transparent audit trail of all analytic decisions. Confirmability was strengthened through reflexive journaling and collaborative interpretation. Transferability was facilitated by providing detailed contextual descriptions of the study setting, participants, and analytic processes, enabling readers to assess the relevance of findings to other contexts.

### 2.9. Ethical Considerations

Ethical approval was obtained from the Institutional Review Board of King Saud University, Riyadh, Saudi Arabia (Reference No: KSU-IRB-2026-0330, 1 April 2026). All procedures were conducted in accordance with the Declaration of Helsinki and relevant institutional guidelines.

Participation was voluntary, and written informed consent was obtained from all participants before data collection. Participants were informed of their right to withdraw at any time without consequence. Identifying information was removed from transcripts, and pseudonyms were assigned to ensure confidentiality. Audio files and transcripts were stored securely on password-protected devices accessible only to the research team. No patient-identifiable information was collected during interviews.

## 3. Results

### 3.1. Participant Characteristics

A total of 18 pediatric nurses participated in the study. Participants were recruited from general pediatric wards, pediatric intensive care units, and neonatal units within tertiary hospitals in Riyadh City. The majority were female (*n* = 14, 77.8%), with ages ranging from 25 to 46 years. The mean age was 32.4 (±5.8) years. Most participants held a bachelor’s degree in nursing (*n* = 12, 66.7%), while six nurses (33.3%) had completed postgraduate education (Master’s degree or specialty certification in pediatric nursing). Participants’ total nursing experience ranged from 2 to 22 years, with a mean of 8.7 (±5.2) years. Pediatric-specific experience ranged from 1 to 16 years, with a mean of 5.9 (±3.8) years. Participants represented six nationalities: Filipino (*n* = 6), Indian (*n* = 4), Saudi (*n* = 4), Egyptian (*n* = 2), Jordanian (*n* = 1), and Sudanese (*n* = 1). The total interview time was 894 min, with individual interviews lasting between 40 and 68 min (mean 49.7 ± 7.2 min). Participant characteristics are summarized in Table 1.

### 3.2. Thematic Findings

Reflexive thematic analysis yielded four interrelated themes that capture how pediatric nurses experienced providing culturally sensitive care to children and their families. These themes reflect the interpretive, communicative, ethical, and emotional dimensions of cross-cultural caregiving. The four themes were: (1) Navigating Cultural Landscapes; (2) Communication as a Bridge and a Barrier; (3) Balancing Professional Standards with Cultural Respect; and (4) The Emotional Labor of Cross-Cultural Caregiving. Together, the findings illuminate the complex professional labor involved in navigating cultural diversity while maintaining compassionate, family-centered pediatric care. Table 2 summarizes the themes, sub-themes, and brief descriptions.

#### 3.2.1. Navigating Cultural Landscapes

This theme captures how nurses encountered and made sense of the wide range of cultural health beliefs, practices, and family structures that shaped pediatric care encounters. Participants described cultural diversity not as an abstract concept but as a daily reality that required ongoing interpretive work.

##### Encountering Diverse Health Beliefs

Participants frequently described encounters with families whose beliefs about illness causation and appropriate treatment differed from biomedical explanations. Traditional remedies, spiritual practices, and folk healing were common, particularly among families from South Asian and African backgrounds.

*“Some families believe the child is sick because of the evil eye. They bring herbs and oils and want to use them alongside our treatment. You have to respect that but also keep the child safe.”*

*(N3, Filipino, General Pediatric Ward, 7 years)*

Several nurses described families who attributed childhood illness to supernatural causes, leading to delays in seeking medical attention or reluctance to accept certain treatments.

*“A mother once told me she didn’t want her baby vaccinated because she believed it would bring bad luck. I had to find a way to explain the importance without dismissing her belief.”*

*(N9, Indian, Neonatal Unit, 5 years)*

Dietary practices also presented challenges, particularly regarding fasting, food restrictions, and nutritional supplementation during illness.

*“During Ramadan, some parents wanted their sick child to fast even though he was on IV fluids. It was a delicate conversation.”*

*(N14, Saudi, PICU, 10 years)*

Across these examples, nurses did not treat cultural and biomedical explanations of illness as mutually exclusive; rather, they worked to hold both systems in view simultaneously, adjusting clinical communication so that biomedical reasoning could be heard without requiring families to abandon their own explanatory framework.

##### Understanding Family Structures and Roles

Cultural norms surrounding family hierarchy, gender roles, and decision-making authority varied considerably across patient populations. Participants described situations where extended family members, rather than parents alone, were involved in care decisions.

*“Sometimes it’s not the mother or father making decisions. The grandmother or the uncle decides everything. You have to know who holds the authority.”*

*(N6, Egyptian, General Pediatric Ward, 8 years)*

Gender-related dynamics also influenced care provision. Several nurses reported situations where fathers preferred male providers for their daughters, or where mothers were reluctant to communicate with male nurses.

*“In some families, the mother won’t speak to me directly because I am male. She communicates through her husband. I have learned to work within those boundaries.”*

*(N11, Jordanian, General Pediatric Ward, 12 years)*

Across these accounts, cultural interpretation functioned less as the application of fixed cultural knowledge than as an ongoing, case-by-case reading of family authority structures and belief systems, requiring nurses to continually revise their assumptions rather than rely on generalized cultural scripts.

#### 3.2.2. Communication Challenges and Facilitators in Cross-Cultural Care

Communication emerged as the most consistently cited challenge and facilitator in cross-cultural pediatric care. Language differences, non-verbal communication patterns, and the adequacy of interpreter services were central to nurses’ experiences.

##### Navigating Language Differences

Language barriers were described as the most immediate and pervasive challenge, affecting assessment accuracy, health education, informed consent processes, and emotional support.

*“When you can’t explain the procedure to the parents or understand their questions, it creates anxiety for everyone: the family, the child, and the nurse.”*

*(N1, Filipino, PICU, 6 years)*

*Nurses reported using a variety of strategies to overcome language barriers, including gestures, translation applications, bilingual colleagues, and visual aids. However, these workarounds were often perceived as inadequate for conveying nuanced clinical information.*

*“Google Translate helps with simple things, but when you need to explain a complex diagnosis or a surgical consent, you need a proper interpreter. We don’t always have that.”*

*(N7, Indian, General Pediatric Ward, 4 years)*

The absence of formal interpreter services was identified as a significant institutional gap that placed nurses in the position of improvising linguistic solutions.

This improvisation reveals a gap between the formal expectation of informed consent and the practical means available to secure it, suggesting that language access functioned as a structural constraint on care quality rather than an incidental inconvenience.

##### Building Trust Through Relational Communication

Despite language challenges, participants emphasized that trust could be established through non-verbal communication, empathetic presence, and small culturally attuned gestures.

*“A smile, a gentle touch on the child’s hand, showing the mother that you care: these things cross all languages.”*

*(N4, Filipino, Neonatal Unit, 9 years)*

Nurses who had worked in Saudi Arabia for extended periods described developing cultural literacy through repeated interactions, learning common Arabic phrases, and understanding unspoken cultural expectations.

*“Over the years, I’ve learned when to give space and when to be close. Some cultures want you present constantly; others prefer privacy. You learn to read the room.”*

*(N13, Indian, PICU, 11 years)*

This pattern suggests that trust in cross-cultural encounters was built less through formal language competence than through relational attentiveness, indicating that institutional responses focused solely on translation technology may address only part of the communicative barrier nurses described.

#### 3.2.3. Negotiating Professional Standards and Cultural Expectations

This theme reflects the ethical and professional tension nurses experienced when cultural practices conflicted with evidence-based care protocols. Participants described a continual process of negotiation between honoring cultural preferences and fulfilling their professional obligations.

##### Negotiating Clinical and Cultural Expectations

Nurses frequently encountered situations requiring them to balance clinical recommendations with culturally rooted expectations from families.

*“A family wanted to use honey on their newborn’s umbilical cord. I knew it wasn’t recommended, but I also knew it was important to them. I explained the risks calmly and offered alternatives.”*

*(N5, Saudi, Neonatal Unit, 6 years)*

Participants described negotiation rather than confrontation as the preferred approach, recognizing that rigid insistence on biomedical protocols could damage the therapeutic relationship.

*“If you dismiss their practices outright, you lose them. They won’t trust you. It’s better to find middle ground where you can.”*

*(N10, Filipino, General Pediatric Ward, 8 years)*

In each case, negotiation depended on nurses first identifying which element of a cultural practice carried symbolic or relational importance for the family, separate from the practice’s clinical risk, so that the two could be addressed independently rather than as an all-or-nothing choice.

##### Advocating for the Child Within Cultural Contexts

When cultural practices posed potential harm to the child, nurses described a heightened sense of advocacy and moral responsibility.

*“When a practice could hurt the child, I have to speak up. That’s non-negotiable. But how I say it matters. I try to be respectful but firm.”*

*(N2, Saudi, PICU, 14 years)*

Child welfare was consistently identified as the overriding priority, even when advocating for the child required navigating complex familial and cultural dynamics.

*“You are the child’s advocate first. Sometimes the family doesn’t understand why we are insisting on something, but you keep explaining until they see it’s for the child’s benefit.”*

*(N16, Egyptian, PICU, 9 years)*

Taken together, these accounts indicate that nurses positioned child welfare, rather than either cultural accommodation or procedural compliance, as the fixed point around which negotiation occurred, a stance that allowed flexibility in method while preserving a consistent ethical boundary.

#### 3.2.4. Emotional Experiences of Cross-Cultural Caregiving

This theme captures the emotional dimensions of providing care across cultural boundaries. Participants described cross-cultural caregiving as emotionally demanding work that involved managing frustration, navigating misunderstandings, and cultivating resilience.

##### Feelings of Frustration and Inadequacy

Nurses described moments of frustration when cultural barriers led to miscommunication, care delays, or perceived non-compliance.

*“There are days when nothing works. You can’t communicate, the family is upset, the child is crying, and you feel helpless.”*

*(N8, Filipino, General Pediatric Ward, 3 years)*

Feelings of inadequacy were particularly acute among less experienced nurses and those new to working in Saudi Arabia.

*“When I first arrived, I felt overwhelmed. I didn’t understand the culture, the language, or what the families expected from me. It took time to adjust.”*

*(N15, Indian, Neonatal Unit, 2 years)*

Notably, participants attributed this early inadequacy less to a deficit in clinical skill than to an absence of structured orientation to the cultural dimensions of practice, pointing to a preventable gap in institutional onboarding rather than an inevitable feature of adjustment.

##### Growth Through Cultural Encounters

Despite the challenges, participants described cross-cultural experiences as opportunities for personal and professional growth. Over time, repeated encounters with diverse families fostered cultural humility, empathy, and adaptability.

*“Every family teaches you something new. I am a better nurse now because of the different cultures I’ve worked with.”*

*(N12, Filipino, General Pediatric Ward, 15 years)*

Several nurses described developing a deeper understanding of their own cultural assumptions through encounters with patients from different backgrounds.

*“Working here has changed how I see things. I used to think my way was the right way. Now I understand there are many right ways.”*

*(N18, Saudi, PICU, 7 years)*

This trajectory from frustration to cultural humility suggests that emotional labor in cross-cultural care was not merely a cost to be managed but, for many participants, a mechanism through which cultural learning itself occurred.

Together, these four themes reveal that providing culturally sensitive care in pediatric nursing involves far more than knowledge of cultural practices; it requires ongoing interpretive engagement, relational skill, ethical negotiation, and emotional resilience.

### 3.3. Conceptual Model of the Study Findings

**Figure 1:**
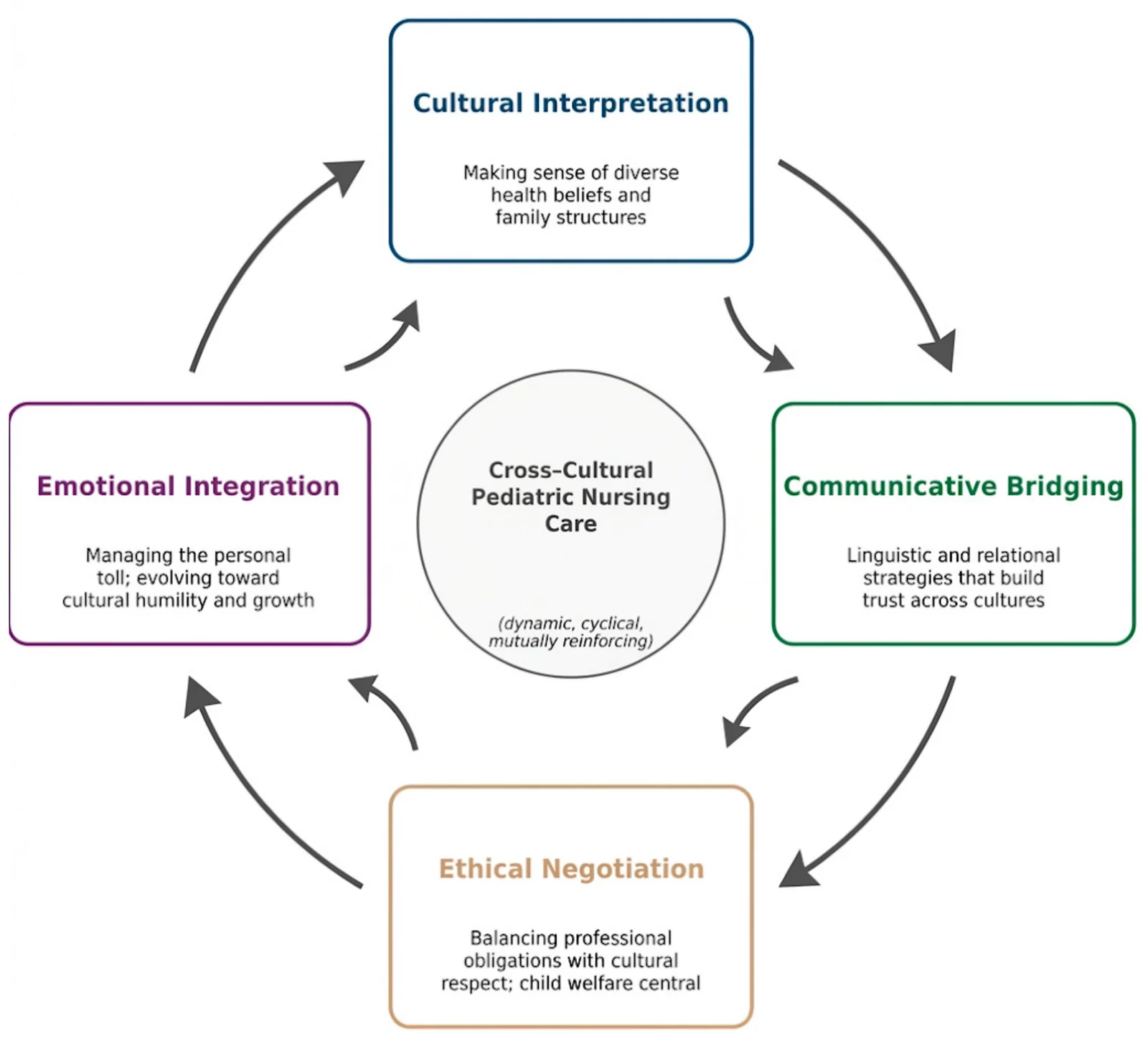
Conceptual model of cross-cultural pediatric nursing care, illustrating the dynamic interplay between cultural interpretation, communicative bridging, ethical negotiation, and emotional integration in providing culturally sensitive care to children and their families. Curved arrows represent the dynamic, cyclical, and mutually reinforcing relationships among the domains, while distinct border colors serve solely for visual differentiation of the themes.

The study findings can be conceptualized as a dynamic model of cross-cultural pediatric nursing care comprising four interconnected dimensions: cultural interpretation, communicative bridging, ethical negotiation, and emotional integration (Figure 1). This model was developed inductively from the four themes generated through the reflexive thematic analysis described in Section 2.7, and represents the authors’ interpretive synthesis of relationships across the coded data rather than a predetermined theoretical framework applied to the findings. Cultural interpretation involves nurses making sense of diverse health beliefs, family structures, and care expectations. Communicative bridging encompasses both linguistic strategies and relational approaches through which trust is established across cultural boundaries. Ethical negotiation captures the ongoing process of balancing professional obligations with cultural respect, with child welfare as the central guiding principle. Emotional integration reflects nurses’ management of the personal and professional toll of cross-cultural care, evolving from frustration toward cultural humility and growth. The model positions these dimensions as cyclical and mutually reinforcing: cultural encounters inform communication strategies, communication shapes ethical negotiations, ethical experiences carry emotional weight, and emotional processing deepens cultural understanding. In the context of Riyadh’s diverse healthcare environment, this model highlights that culturally sensitive pediatric nursing is not a static competence but a dynamic, experiential practice refined through sustained cross-cultural engagement.

## 4. Discussion

This study explored how pediatric nurses in Riyadh, Saudi Arabia experience providing culturally sensitive care to children and their families. Building on the four themes presented above, this section situates nurses’ experiences within existing theoretical and empirical literature on cultural competence, communication, and emotional labor in nursing. Nurses described cultural encounters as dynamic, contextually situated interactions requiring continuous adaptation, relational skill, and professional judgment. Importantly, cultural sensitivity emerged not as a fixed competence but as an evolving practice shaped by experience and refined through sustained engagement with diverse populations.

### 4.1. Interpretation in Relation to Existing Literature

#### 4.1.1. Navigating Cultural Landscapes: Cultural Interpretation in Practice

The findings align with and extend existing scholarship on cultural competence in nursing. Leininger’s theory of Culture Care Diversity and Universality [26] posits that understanding cultural meanings embedded in care practices is essential for providing effective nursing interventions. Participants in the present study similarly described the need to interpret families’ cultural health beliefs, not as obstacles to be overcome, but as meaningful frameworks within which parents understood and responded to their child’s illness. However, our findings extend Leininger’s framework by foregrounding the negotiative and emotionally demanding dimensions of cross-cultural care that may be under-addressed in theoretical models. This gap is consistent with more recent qualitative findings that qualified nurses in multiple European countries continue to describe cultural competence as an evolving, situational skill shaped by direct exposure to diverse patients, rather than a static attribute acquired through training alone [27].

#### 4.1.2. Communication as a Bridge and a Barrier

Communication emerged as the most salient challenge, consistent with international literature identifying language barriers as the primary impediment to culturally competent care [28,29]. Participants described language differences as affecting not only clinical communication but also trust-building, emotional support, and family participation in care decisions. This finding resonates with research documenting that inadequate language support compromises patient safety and satisfaction in multicultural healthcare settings [30]. It is also consistent with a recent qualitative study of nurse-parent communication in culturally sensitive pediatric care, which similarly identified language and cultural discordance as central barriers to trust-building [15]. The institutional absence of formal interpreter services was particularly notable, reflecting a systemic gap that placed the burden of linguistic mediation on individual nurses.

#### 4.1.3. Balancing Professional Standards with Cultural Respect

The tension between evidence-based practice and cultural accommodation has been described in previous research on cross-cultural care [31,32]. Participants in this study described using negotiation rather than confrontation when cultural practices conflicted with clinical recommendations, an approach consistent with the concept of cultural negotiation in transcultural nursing theory [33]. Importantly, child welfare was consistently identified as the overriding ethical priority, reflecting nurses’ role as advocates for vulnerable pediatric patients within complex familial and cultural contexts. A similar negotiation-based stance, balancing cultural accommodation against clinical protocol, has recently been reported among nurses in high-acuity critical care settings, suggesting that this approach may generalize beyond pediatric practice [34].

#### 4.1.4. The Emotional Labor of Cross-Cultural Caregiving

The emotional dimensions of cross-cultural care identified in this study align with Hochschild’s concept of emotional labor [35], extended to the nursing context by researchers examining the affective demands of culturally diverse practice [36]. Participants described frustration, inadequacy, and emotional exhaustion as recurring experiences, particularly early in their careers. However, the finding that cross-cultural encounters also fostered growth, humility, and empathy adds nuance to the literature that has predominantly framed cultural diversity as a source of professional burden. This growth trajectory is consistent with the concept of cultural humility [37], which emphasizes ongoing self-reflection and openness to learning rather than mastery of cultural knowledge. This extends beyond how emotional labor has typically been conceptualized in nursing [35,36], where the caregiver’s own cultural background is rarely treated as directly relevant to the labor itself; in the present study, nurses’ cultural identity was inseparable from how they interpreted, managed, and drew meaning from emotionally demanding cross-cultural encounters.

#### 4.1.5. Toward an Integrated Model of Cross-Cultural Pediatric Care

The unique bidirectional cultural diversity of the Saudi healthcare context, in which both patients and nurses represent multiple nationalities, adds complexity not fully captured by Western-derived cultural competence models. Previous research in Gulf Cooperation Council countries has documented similar dynamics [38,39], but qualitative exploration of how nurses subjectively experience and navigate this complexity in pediatric settings has been limited. This is consistent with emerging international evidence that culturally and linguistically diverse nurses themselves require structured organizational support to integrate into multinational care teams [17], suggesting that bidirectional cultural diversity shapes not only patient care but also workforce cohesion. The present study contributes contextualized insights relevant to healthcare systems characterized by multinational workforces and patient populations. The specific contribution of the Saudi Arabian context therefore lies in the coexistence of these two axes of diversity within a single, rapidly expanding healthcare system shaped by Vision 2030’s international workforce recruitment strategy, a configuration that existing cultural competence and emotional labor frameworks, largely developed with a single axis of diversity in view, do not fully anticipate. Read together, the four themes discussed above (cultural interpretation, communicative bridging, ethical negotiation, and emotional integration) are not independent findings but interlocking dimensions of a single practice; Section 3.3 develops this integration explicitly as a conceptual model of cross-cultural pediatric nursing care.

### 4.2. Implications for Practice, Education, and Policy

The findings carry several implications for pediatric nursing practice, education, and policy. First, healthcare institutions should invest in formal interpreter services and multilingual patient education materials to reduce the communicative burden on individual nurses. Technology-assisted translation tools, while useful for basic interactions, are insufficient for complex clinical communication and should complement, not replace, professional interpretation services.

Second, cultural competence education for pediatric nurses should move beyond factual knowledge of cultural practices toward experiential, reflective, and simulation-based approaches that develop cultural humility, negotiation skills, and emotional resilience. Integrating case-based learning scenarios reflecting the cultural diversity of the local patient population may better prepare nurses for the complexities of cross-cultural care encounters.

Third, organizational policies should recognize the emotional labor of cross-cultural caregiving and provide structured support, including peer debriefing, clinical supervision, and mentorship programs that facilitate sharing of cross-cultural experiences. Creating a culture of openness toward cultural differences within multidisciplinary teams may reduce professional isolation and enhance collaborative care.

Fourth, at a policy level, the findings support the integration of cultural competence standards into nursing licensure and continuing education requirements in Saudi Arabia, aligned with Vision 2030’s emphasis on healthcare quality and patient-centered service delivery [40]. For nursing management specifically, these findings suggest a role for unit and department leaders in workforce allocation (for example, considering language and cultural background alongside clinical competence when assigning nurses to complex cases), in formally recognizing interpreter-mediated and culturally negotiated care as demanding professional labor rather than routine practice, and in modeling the reflective, non-judgmental stance toward cultural difference that participants associated with professional growth.

### 4.3. Strengths and Limitations

This study provides in-depth, contextually grounded insights into cross-cultural pediatric nursing care in Riyadh, Saudi Arabia, a rapidly developing healthcare context with significant cultural diversity. The qualitative descriptive design enabled rich exploration of nurses’ experiences, including tacit knowledge and emotional dimensions that may not be captured through quantitative methodologies.

However, limitations should be acknowledged. Findings are based on self-reported experiences from nurses in tertiary hospitals within one city, which may limit transferability to other healthcare settings or regions. Participants’ accounts may be influenced by recall bias or social desirability. Although reflexive practices were employed throughout, complete elimination of researcher pre-understandings was neither possible nor intended within the qualitative descriptive framework. The study focused exclusively on nurses’ perspectives; incorporating the views of families and children would provide a more comprehensive understanding of cross-cultural care dynamics. Future research across diverse settings, incorporating observational methods and family perspectives, is recommended.

## 5. Conclusions

This study reveals that providing culturally sensitive care to children and their families is a complex, dynamic, and emotionally demanding practice that involves ongoing cultural interpretation, communicative adaptation, ethical negotiation, and emotional resilience. In direct response to the study’s three research questions, nurses described their experiences through four intertwined themes rather than a single dominant narrative (Research Question 1); the principal challenge identified was language and communication difficulty compounded by the absence of formal interpreter services, while the principal facilitators were relational, non-verbal communication strategies and a negotiation-based rather than confrontational stance toward cultural difference (Research Question 2); and nurses negotiated cultural differences by treating the child’s welfare as a fixed ethical boundary within which culturally responsive flexibility was exercised (Research Question 3). Pediatric nurses in Riyadh’s diverse healthcare environment engage in sophisticated professional labor that extends beyond clinical competence to encompass relational, moral, and emotional dimensions of care. The findings underscore the need for institutional investment in interpreter services, culturally responsive education, and organizational support structures that sustain nurses’ capacity to provide equitable, compassionate, and culturally attuned pediatric care. Future research should examine the effectiveness of culturally tailored interventions and explore the experiences of families receiving cross-cultural pediatric care.

## Figures and Tables

**Table 1:** Socio-demographic characteristics of participants (N = 18).

Socio-Demographics	Frequency	Percentage
Mean (SD) age	32.4 (5.8) years	N/A
Gender		
Male	4	22.2%
Female	14	77.8%
Educational level		
Bachelor’s degree	12	66.7%
Postgraduate degree	6	33.3%
Mean (SD) total nursing experience	8.7 (5.2) years	N/A
Mean (SD) pediatric experience	5.9 (3.8) years	N/A
Nationality		
Filipino	6	33.3%
Indian	4	22.2%
Saudi	4	22.2%
Egyptian	2	11.1%
Jordanian	1	5.6%
Sudanese	1	5.6%
Pediatric unit type		
General pediatric ward	8	44.4%
Pediatric ICU	6	33.3%
Neonatal unit	4	22.2%

**Table 2:** Themes, sub-themes, and descriptions.

Theme	Sub-Theme	Description
3.2.1 Navigating Cultural Landscapes	Section Encountering Diverse Health Beliefs	Nurses encountered varying beliefs about illness causation, traditional remedies, and spiritual healing that shaped family expectations and care preferences.
	Section Understanding Family Structures and Roles	Cultural norms regarding parental authority, gender roles, and extended family involvement influenced care dynamics and decision-making.
3.2.2 Communication as a Bridge and a Barrier	Section Navigating Language Differences	Language barriers created misunderstandings and limited nurses’ ability to assess, educate, and build rapport with families.
	Section Building Trust Through Relational Communication	Non-verbal cues, empathetic presence, and culturally attuned engagement facilitated therapeutic relationships.
3.2.3 Balancing Professional Standards with Cultural Respect	Section Negotiating Clinical and Cultural Expectations	Nurses experienced tension between evidence-based protocols and family cultural practices, requiring negotiation and flexibility.
	Section Advocating for the Child Within Cultural Contexts	Nurses prioritized child welfare while respecting family autonomy, navigating ethical complexity.
3.2.4 The Emotional Labor of Cross-Cultural Caregiving	Section Feelings of Frustration and Inadequacy	Repeated cultural miscommunication and perceived inability to meet diverse needs generated emotional strain.
	Section Growth Through Cultural Encounters	Over time, cross-cultural experiences fostered professional growth, empathy, and cultural humility.

## Data Availability

The qualitative datasets analyzed during the current study are not publicly available due to confidentiality agreements with participants but are available from the corresponding author upon reasonable request. Audio recordings and transcripts are stored securely and retained in accordance with the data retention requirements of the King Saud University Data Management Office, after which they will be securely destroyed; participants were informed of this retention arrangement as part of the consent process.

## Supplementary Materials

The following supporting information can be downloaded at https://www.mdpi.com/article/10.3390/healthcare14172784/s1.

## Abbreviations

The following abbreviations are used in this manuscript:

COREQ	Consolidated Criteria for Reporting Qualitative Research
IRB	Institutional Review Board
ORF	Ongoing Research Funding Program
PICU	Pediatric Intensive Care Unit
SRQR	Standards for Reporting Qualitative Research

## References

[1] Bhopal R.S. (2014). Migration, Ethnicity, Race, and Health in Multicultural Societies.

[2] Vision 2030 Kingdom of Saudi Arabia (2021). Health Sector Transformation Program.

[3] Coyne I., Hallström I., Söderbäck M. (2016). Reframing the focus from a family-centered to a child-centered care approach for children’s healthcare. J. Child Health Care.

[4] Campinha-Bacote J. (2002). The process of cultural competence in the delivery of healthcare services: A model of care. J. Transcult. Nurs..

[5] Shen Z. (2015). Cultural competence models and cultural competence assessment instruments in nursing: A literature review. J. Transcult. Nurs..

[6] Betancourt J.R., Green A.R., Carrillo J.E., Ananeh-Firempong O. (2003). Defining cultural competence: A practical framework for addressing racial/ethnic disparities in health and health care. Public Health Rep..

[7] Power N., Franck L. (2008). Parent participation in the care of hospitalized children: A systematic review. J. Adv. Nurs..

[8] Cai D. (2016). A concept analysis of cultural competence. Int. J. Nurs. Sci..

[9] Almutairi A.F., McCarthy A., Gardner G.E. (2015). Understanding cultural competence in a multicultural nursing workforce: Registered nurses’ experience in Saudi Arabia. J. Transcult. Nurs..

[10] Shields L., Zhou H., Pratt J., Taylor M., Hunter J., Pascoe E. (2012). Family-centered care for hospitalized children aged 0–12 years. Cochrane Database Syst. Rev..

[11] Alsadaan N., Jones L.K., Kimpton A., DaCosta C. (2021). Challenges facing the nursing profession in Saudi Arabia: An integrative review. Nurs. Rep..

[12] Al-Khathami A.M., Kojan S.W., Aljumah M.A., Alqahtani H., Alrwaili H. (2010). The effect of nurse-patient language barrier on patients’ satisfaction. Saudi Med. J..

[13] Albougami A.S., Alotaibi J.S., Alsharari A.F., Albagawi B.S., Almazan J.U., Maniago J.D., EiRazkey J.Y. (2019). Cultural competence and perception of patient-centered care among non-Muslim expatriate nurses in Saudi Arabia: A cross-sectional study. Pak. J. Med. Health Sci..

[14] Cruz J.P., Estacio J.C., Bagtang C.E., Colet P.C. (2016). Predictors of cultural competence among nursing students in the Philippines: A cross-sectional study. Nurse Educ. Today.

[15] Tavallali A.G., Jirwe M., Johansson Stark Å., Eckerblad J. (2025). Factors influencing nurse-to-parent communication in culturally sensitive pediatric care: A qualitative study. J. Pediatr. Nurs..

[16] Golsäter M., Karlsson Fiallos M., Olsson Vestvik S., Anefur H., Harder M. (2023). Child health care nurses’ cultural competence in health visits with children of foreign background. Nurs. Open.

[17] Kamau S., Oikarainen A., Kiviniitty N., Koskenranta M., Kuivila H., Tomietto M., Kanste O., Mikkonen K. (2023). Nurse leaders’ experiences of how culturally and linguistically diverse registered nurses integrate into healthcare settings: An interview study. Int. J. Nurs. Stud..

[18] Creswell J.W., Poth C.N. (2018). Qualitative Inquiry and Research Design: Choosing Among Five Approaches.

[19] Sandelowski M. (2000). Whatever happened to qualitative description?. Res. Nurs. Health.

[20] Braun V., Clarke V. (2019). Reflecting on reflexive thematic analysis. Qual. Res. Sport Exerc. Health.

[21] Tong A., Sainsbury P., Craig J. (2007). Consolidated criteria for reporting qualitative research (COREQ): A 32-item checklist for interviews and focus groups. Int. J. Qual. Health Care.

[22] Saunders B., Sim J., Kingstone T., Baker S., Waterfield J., Bartlam B., Burroughs H., Jinks C. (2018). Saturation in qualitative research: Exploring its conceptualization and operationalization. Qual. Quant..

[23] Malterud K., Siersma V.D., Guassora A.D. (2016). Sample size in qualitative interview studies: Guided by information power. Qual. Health Res..

[24] O’Brien B.C., Harris I.B., Beckman T.J., Reed D.A., Cook D.A. (2014). Standards for reporting qualitative research: A synthesis of recommendations. Acad. Med..

[25] Lincoln Y.S., Guba E.G. (1985). Naturalistic Inquiry.

[26] Leininger M., McFarland M.R. (2002). Transcultural Nursing: Concepts, Theories, Research and Practice.

[27] Antón-Solanas I., Rodríguez-Roca B., Vanceulebroeck V., Kömürcü N., Kalkan I., Tambo-Lizalde E., Huércanos-Esparza I., Casa Nova A., Hamam-Alcober N., Coelho M. (2022). Qualified nurses’ perceptions of cultural competence and experiences of caring for culturally diverse patients: A qualitative study in four European countries. Nurs. Rep..

[28] Gerchow L., Burka L.R., Miner S., Squires A. (2021). Language barriers between nurses and patients: A scoping review. Patient Educ. Couns..

[29] Al Shamsi H., Almutairi A.G., Al Mashrafi S., Al Kalbani T. (2020). Implications of language barriers for healthcare: A systematic review. Oman Med. J..

[30] Flores G. (2005). The impact of medical interpreter services on the quality of health care: A systematic review. Med. Care Res. Rev..

[31] Almutairi A.F., Adlan A.A., Nasim M. (2017). Perceptions of the critical cultural competence of registered nurses in Canada. BMC Nurs..

[32] Dreher M., MacNaughton N. (2002). Cultural competence in nursing: Foundation or fallacy?. Nurs. Outlook.

[33] Purnell L. (2002). The Purnell model for cultural competence. J. Transcult. Nurs..

[34] Červený M., Balounová L., Kratochvílová I., Doležalová J., Tóthová V. (2024). Perceptions of nurses on the scope of culturally competent care in critical care: A qualitative study. Nurs. Crit. Care.

[35] Hochschild A.R. (2012). The Managed Heart: Commercialization of Human Feeling.

[36] Gray B., Smith P. (2009). Emotional labor and the clinical settings of nursing care: The perspectives of nurses in East London. Nurse Educ. Pract..

[37] Tervalon M., Murray-García J. (1998). Cultural humility versus cultural competence: A critical distinction in defining physician training outcomes in multicultural education. J. Health Care Poor Underserved.

[38] Almalki M., FitzGerald G., Clark M. (2011). Health care system in Saudi Arabia: An overview. East Mediterr. Health J..

[39] El-Haddad M. (2006). Nursing in the United Arab Emirates: An historical background. Int. Nurs. Rev..

[40] Alkhamis A., Hassan A., Cosgrove P. (2014). Financing healthcare in Gulf Cooperation Council countries: A focus on Saudi Arabia. Int. J. Health Plan. Manag..

