# Supplementary material for: Cross-Cultural Care: The Experiences of Pediatric Nurses in Providing Culturally Sensitive Care to Children and Their Families
Source: Healthcare (Basel). 2026 Sep 1;14(17):2784. doi: 10.3390/healthcare14172784 (PMC13566556; doi:10.3390/healthcare14172784)
Supplement: Supplementary file 1 [file healthcare-14-02784-s001.zip › healthcare-4469422-supplementary file S1.pdf]

COREQ (Consolidated Criteria for Reporting Qualitative Research) 32-Item Checklist

Item	Guide question / topic	Reported on page/section
	**Domain 1: Research team and reflexivity — Personal characteristics**	
1	Interviewer/facilitator	§2.6 Researcher Characteristics and Reflexivity
2	Credentials	Title page / author affiliations
3	Occupation	Title page / author affiliations
4	Gender	Reported
5	Experience and training	§2.6
	Relationships with participants	
6	Relationship established	§2.4 Recruitment; §2.6
7	Participant knowledge of interviewer	§2.4
8	Interviewer characteristics	§2.6
	**Domain 2: Study design — Theoretical framework**	
9	Methodological orientation and theory	§2.1 Design
	Participant selection	
10	Sampling	§2.3 Participants and Sampling
11	Method of approach	§2.4 Recruitment
12	Sample size	§2.3; §3.1 (N = 18)
13	Non-participation	§2.3 (three nurses approached declined, citing scheduling conflicts)
	Setting	
14	Setting of data collection	§2.2 Setting; §2.5 Data Collection
15	Presence of non-participants	§2.5 (private room / secure video conferencing)
16	Description of sample	§3.1 Participant Characteristics; Table 1
	Data collection	
17	Interview guide	§2.5; Supplementary File 1
18	Repeat interviews	§2.5 (no repeat interviews; each participant interviewed once)
19	Audio/visual recording	§2.5 (audio-recorded, transcribed verbatim)
20	Field notes	§2.5
21	Duration	§2.5; §3.1 (mean 49.7 ± 7.2 min)
22	Data saturation	§2.3 (data richness and thematic sufficiency, ref. 19)
23	Transcripts returned	§2.8 (theme-summary member checking)
	**Domain 3: Analysis and findings — Data analysis**	
24	Number of data coders	§2.7 (two authors coded the data manually)
25	Description of the coding tree	§2.7 Data Analysis
26	Derivation of themes	§2.7; §3.2
27	Software	§2.7 (manual coding; no software used)
28	Participant checking	§2.8
	Reporting	
29	Quotations presented	§3.2.1–3.2.4
30	Data and findings consistent	§3.2–3.3
31	Clarity of major themes	§3.2 Table 2; §3.3 Conceptual Model
32	Clarity of minor themes	§3.2.1–3.2.4 sub-themes
